# CAR T Cells for Acute Myeloid Leukemia: State of the Art and Future Directions

**DOI:** 10.3389/fonc.2020.00697

**Published:** 2020-05-06

**Authors:** Sherly Mardiana, Saar Gill

**Affiliations:** ^1^Center for Cellular Immunotherapies, University of Pennsylvania School of Medicine, Philadelphia, PA, United States; ^2^Division of Hematology-Oncology and Center for Cellular Immunotherapies, University of Pennsylvania, PA, United States

**Keywords:** chimeric antigen receptor, acute myeloid leukemia, engineered T cells, adoptive therapy, immunotherapy

## Abstract

Relapse after conventional chemotherapy remains a major problem in patients with myeloid malignancies such as acute myeloid leukemia (AML), and the major cause of death after diagnosis of AML is from relapsed disease. The only potentially curative treatment option currently available is allogeneic hematopoietic stem cell transplantation (allo-HSCT), which through its graft-vs.-leukemia effects has the ability to eliminate residual leukemia cells. Despite its long history of success however, relapse following allo-HSCT is still a major challenge and is associated with poor prognosis. In the field of adoptive therapy, CD19-targeted chimeric antigen receptor (CAR) T cells have yielded remarkable clinical success in certain types of B-cell malignancies, and substantial efforts aimed at translating this success to myeloid malignancies are currently underway. While complete ablation of CD19-expressing B cells, both cancerous and healthy, is clinically tolerated, the primary challenge limiting the use of CAR T cells in myeloid malignancies is the absence of a dispensable antigen, as myeloid antigens are often co-expressed on normal hematopoietic stem/progenitor cells (HSPCs), depletion of which would lead to intolerable myeloablation. This review provides a discussion on the current state of CAR T cell therapy in myeloid malignancies, limitations for clinical translation, as well as the most recent approaches to overcome these barriers, through various genetic modification and combinatorial strategies in an attempt to make CAR T cell therapy a safe and viable option for patients with myeloid malignancies.

## Introduction

Myeloid malignancies are clonal diseases of the hematopoietic stem or progenitor cells (HSPCs) that arise from genetic and/or epigenetic changes, resulting in deleterious effects on critical pathways such as cell differentiation, proliferation, and self-renewal. These malignancies can be categorized into myelodysplastic syndromes (MDS), myeloproliferative neoplasms (MPN), MDS/MPN such as chronic myelomonocytic leukemia (CMML), and acute myeloid leukemia (AML) (1). Currently, the only therapeutic modality that is potentially curative in hematological malignancies is allogeneic hematopoietic stem cell transplantation (allo-HSCT). In addition to anti-tumor effects from chemotherapy and/or total body irradiation given to patients prior to allo-HSCT, the overall therapeutic effect of allo-HSCT also relies on the graft-vs.-leukemia (GvL) phenomenon, that occurs when donor T cells recognize foreign antigens on the host's hematopoietic tissues and subsequently mediate tumor elimination (2). This GvL phenomenon demonstrates the responsiveness of myeloid diseases to T cells. However, despite the successful history of HSCT with ~20,000 people undergoing HSCT annually in the US alone (3), relapse following allo-HSCT is still a major challenge and is associated with poor prognosis.

Over the years, a plethora of immunotherapy-based treatment approaches for hematological malignancies have been developed and tested in both pre-clinical and clinical settings. Among these, the most striking progress has occurred in B-lymphoid malignancies such as acute lymphocytic leukemia (ALL) and certain lymphomas, where immunotherapy agents such as chimeric antigen receptor (CAR) T cells, bispecific T cell engagers (BiTEs), and immune checkpoint blockade (ICB) particularly in Hodgkin lymphoma, have shown robust clinical responses (4–7). Building on these successes, substantial efforts have been directed toward translating these approaches to treating myeloid malignancies. BiTEs recognizing the T cell molecule CD3 and a myeloid cell antigen such as CLL-1, CD123, or CD33 are under investigation (8). The use of ICB as monotherapy in AML and MDS has unfortunately shown only modest clinical responses, and ICB is now being tested in combination with hypomethylating agents (HMAs), which are thought to have immunomodulatory effects including enhancing tumor cell antigen presentation and co-stimulatory molecule expression (9). Indeed, HMAs such as decitabine and azacitidine have been reported to upregulate the expression of several cancer testis antigens such as NY-ESO-1 and MAGE (10, 11).

In the context of cellular therapy, the US Food and Drug Administration (FDA) approval of two CD19-directed CAR T cells products, Kymriah^TM^ and Yescarta^TM^, revolutionized the field of immunotherapy and ignited our enthusiasm for translating this promising technology to the treatment of myeloid malignancies. However, unlike B cell malignancies that express several antigens exclusive to the B cell lineage such as CD19, CD20, CD22, or BCMA in the case of myeloma, most tumor antigens targetable in myeloid malignancies are shared with a wide range of healthy cells including HSPCs. Moreover, whereas B cell aplasia that occurs as a result of B cell antigen-specific CAR T cell therapy is clinically benign and can be managed with infusions of intravenous immunoglobulin, prolonged myeloablation is not feasible due to a risk of infection and transfusion dependence. Herein, we provide an overview of the current state as well as future prospects of CAR T cell therapy options for patients with myeloid malignancies, specifically focusing on AML.

## The Current State of CAR T Cell Therapy in AML

The first reported clinical trial that demonstrated biological activity of CAR T cells in AML was published in 2013 by Ritchie et al. utilizing a second generation CD28-ζ CAR directed against the Lewis Y antigen. Although only limited efficacy was observed, this was an important study given its demonstration of CAR T cell biological activity in AML patients in the absence of overt hematopoietic toxicity (12). Currently, there are more than twenty CAR T cell clinical trials enrolling patients with AML, mostly targeting CLL-1, CD33, or CD123 (13). CD33 and CD123 are both appealing target antigens, given they are both almost ubiquitously expressed on AML blasts, though they are also expressed by healthy HSPCs (14). While mature clinical data have yet to be published, there have been a number of case reports and pilot studies reporting the use of CAR T cells in AML (15–18). For example, a partial response to CD33-specific CAR T cells was described in a case report of a 41-year-old male. The patient had a marked transient reduction of AML blasts in the marrow before the disease progressed at 9 weeks post CAR T cell treatment. A significant cytokine release syndrome (CRS) was observed in this patient (15).

CLL-1 (also known as CLEC12A) is also an attractive target for CAR T cells owing to its high expression in AML and reported absence in healthy HSPCs, though in our hands CLL-1 is detected on all monocytes as well as on some early hematopoietic cells (19). CLL-1 is also known to be rarely expressed on non-hematological cells (20). It has been reported that more than 60% AML samples with CD33 expression also express CLL-1 (21), leading to the idea of creating dual-specific CAR T cells for both antigens. Supported by pre-clinical data showing specificity and anti-tumor potency of dual CD33-CLL-1 CAR T cells, a first-in-human trial using these dual CAR T cells was conducted. A preliminary report by Liu et al. described that two patients with refractory/relapsed (R/R) AML were treated with the dual CAR T cells following fludarabine and cyclophosphamide preconditioning. Both of these patients experienced measurable residual disease (MRD)^−ve^ complete remission accompanied by pancytopenia within 3 weeks of CAR T cell infusion. These patients later underwent anti-thymocyte globulin (ATG)-based HSCT with subsequent hematopoietic recovery (18, 20).

At the University of Pennsylvania, a clinical trial of CD123-specific CAR T cells manufactured via mRNA electroporation was recently completed. This method, rather than lentiviral transduction, was chosen to prevent long-term CAR T cell persistence thus avoiding the risk of severe myeloablation. Patients with R/R AML received lymphodepleting chemotherapy prior to treatment with transiently acting CAR T cells. In this study, although no measurable anti-tumor responses were observed, some level of CAR T cell bioactivity was found, as demonstrated by the cytokine release syndrome (CRS) and/or fever experienced by all treated patients. As expected with mRNA-based CAR T cells, only transient CAR T cell detection *in vivo* was observed. Of note, no overt vascular, hematologic or neurologic toxicity was reported despite expression of the target antigen on healthy hematopoietic tissues and some small-caliber blood vessels (17). This favorable safety profile supported the development of a clinical trial using a lentiviral transduction system (CD123-4-1BB-ζ), which is currently open (*clinicaltrials.gov identifier: 03766126*). In addition, early results from an ongoing trial at the City of Hope Medical Center (*clinicaltrials.gov identifier: 02159495*) were also recently reported. This study employed second generation CD28-ζ CAR T cells targeting CD123 manufactured by lentiviral transduction. Six patients with refractory AML were treated with either 50 or 200 million CAR T cells after being preconditioned with chemotherapy. Results revealed one of the two patients treated with the lower dose of CAR T cells experienced a transient morphologic leukemia-free state. This patient then received a subsequent infusion of CAR T cells 3 months later and achieved blast reduction from 77.9 to 0.9%. Encouragingly, two of the four patients receiving a higher dose of 200 million CAR T cells experienced complete remission and went on to HSCT. The other two patients had transient partial responses. Grade 1 or 2 CRS was reported in most patients, but no dose limiting toxicity including cytopenia was observed at the time of the report (22). As of February 2019, 24 patients were reported to have been enrolled (23), and further results on additional patients from this study are eagerly awaited.

## Limitations of CAR T Cell Therapy in AML and Strategies to Overcome Them

### Lack of a Leukemia-Specific Antigen for Use as Target for CAR T Cells

The fundamental biological barrier limiting the application of CAR T cell therapy in AML is the absence of an AML-specific antigen. AML cells express various cell surface antigens including CD123, CD34, CD33, and many others. However, these same antigens are also shared by healthy HSPCs and their myeloid and/or lymphoid progenitors (24). CD123-directed and CD33-directed CAR T cells have both shown highly potent anti-tumor activity in pre-clinical models (25–27). However, CAR T cells are unable to differentiate between normal and cancerous cells. Unlike in the case of CD19 CAR T cells whereby complete elimination of both normal and cancerous B cells is clinically benign, in stark contrast, prolonged myeloablation as a result of CAR T cells targeting myeloid antigens shared with normal myeloid progenitors is ultimately fatal due to neutropenic infections and bleeding complications. In order to mitigate such toxicities, substantial efforts have been invested in developing and testing a variety of solutions.

#### Limiting CAR T Cell Persistence to Prevent Protracted Myeloablation

One potential strategy to prevent the risk of bone marrow failure following CAR T cells that target the highly expressed but non-leukemia specific antigens such as CD33 and CD123 is to limit long-term CAR T cell persistence *in vivo*. Numerous approaches have been tested both in pre-clinical and clinical studies to incorporate a “safety switch” into T cells, allowing for elimination of T cells *in vivo* if needed. A suicide gene that has long been utilized in T cell therapy is the herpes simplex virus-thymidine kinase (HSV-tk), which allows for selective depletion of expressing cells upon administration of a prodrug. In this case, HSV-tk is able to turn the prodrug into a toxic compound that halts DNA replication, hence resulting in cell death (28). The use of HSV-tk however is limited by immunogenicity of the viral enzyme and the relatively long latency to activation, which is not suitable for managing toxicity that requires immediate termination (29). A more advanced suicide system employs the co-expression of inducible caspase 9 (iCasp9) in T cells. This construct fuses the intracellular domain of caspase 9, a known pro-apoptotic protein, to a drug-binding domain from FK506-binding protein. Administration of a synthetic molecule drug called AP1903 leads to dimerization of the fusion proteins and ultimately rapid ablation of T cells (30, 31). The iCasp9 suicide system was tested clinically in the setting of haploidentical stem cell transplantation (32), and has also been explored in the setting of CAR T cell therapy in pre-clinical study by Hoyos et al. (33). Subsequently, the iCasp9 suicide system has been incorporated in the CAR construct of various clinical trials (*clinicaltrials.gov identifier: 02992210, 01822652*) (29), however to our knowledge AP1903 administration has never been required and hence there is no formal proof of its efficacy in the CAR T cell setting.

Another option for a safety switch is to engineer T cells to co-express a truncated well-characterized surface antigen against which clinically approved monoclonal antibodies exist (for example truncated EGFR targetable by cetuximab, and truncated CD20 targetable by rituximab). This platform could allow antibody-based elimination of the adoptively transferred T cells by complement-dependent cytotoxicity or antibody-dependent cellular cytotoxicity (ADCC) (34). There are also non-specific drugs that eliminate the transferred T cells as well as endogenous T cells, including anti-thymocyte globulin (ATG), or the anti-CD52 monoclonal antibody alemtuzumab (35). A completely different approach to limit CAR T cell persistence that does not require administration of exogenous antibodies is to use mRNA electroporation for incorporation of the CAR into T cells, whereby CAR T cell function is inherently limited due to degradation of the mRNA (17).

It is increasingly apparent that the choice of co-stimulatory domain in the CAR constructs could affect the CAR T cell ability to persist *in vivo*. Comparison between CD28 and 4-1BB has revealed superior persistence of CAR T cells incorporating 4-1BB signaling domain over those with CD28 (36). Although never directly compared in the context of anti-AML CAR T cells, the use of CD28 co-stimulatory domain may lead to shorter CAR T cell persistence and could therefore impact the duration of CAR T cell-induced myeloablation. Notably, accumulating evidence from CD19-directed CAR T cell trials in B-cell leukemia suggests that durable clinical response appears to be associated with CAR T cell expansion and persistence, and failure to persist seems to correlate with increased risk of relapse (37, 38). The optimal duration of CAR T cell persistence for disease response, at least for CD19 CAR T cells, is predicted to be at least 3–6 months (4, 39). Therefore, placing a limit on CAR T cell persistence will likely create a new problem of limiting CAR T cell therapeutic potential and consequently increasing the risk of disease relapse.

#### Creating an Artificial “AML-Specific” Antigen by Genetic Editing of Allograft

A novel strategy to allow for CAR T cell long-term persistence without the unwanted prolonged myeloablation effect is to edit out the CAR target antigen, for example CD33, from a donor allograft. The idea is to transplant CD33^−/−^ HSPCs into the patient. Once engrafted, CD33-specific CAR T cells manufactured from the same donor may then be given to the patient, with the goal of allowing for normal hematopoiesis by the CD33^−/−^ allograft in the presence of continued CAR T cell persistence. Indeed, our group has evaluated this approach in both *in vitro* and *in vivo* studies. Using the CRISPR/Cas9 technology, we demonstrated that CD33^−/−^ HSPCs and their progeny were resistant to CD33-directed CAR T cells in murine xenograft. Importantly, such CD33 deletion did not impair the hematopoietic and immunological function of the HSPCs and their progeny in murine xenograft and in non-human primate models (26). A clinical trial involving the use of allogeneic CD33^−/−^ HSCT prior to CAR T cell infusion is currently being devised at the University of Pennsylvania for patients with R/R AML. During the conduct of the trial, careful assessment of potential side effects will include off-target editing in HSPCs, clinical consequences of CD33 deletion in the bone marrow, as well as the effect of CAR T cells on healthy tissues that may express CD33.

Another potential antigen that may be edited using a similar approach is CD123. However, since CD123 serves a function as the alpha subunit of the IL-3 receptor, complete removal of CD123 in the hematopoietic system is predicted to have a wide range of deleterious effects, given that IL-3 is a pleiotropic cytokine involved in hematopoietic development (40). Thus, an alternative approach could include targeted removal of the epitope on the CD123 molecule that is recognized by the CAR T cells, or to knockdown (instead of completely knockout) CD123 expression in donor HSPCs to a level below the CAR T cell activation threshold, but is still sufficient to preserve normal CD123 signaling and hematopoiesis. This approach is currently under investigation.

#### Identifying Leukemia-Specific Neoantigens

Designing a potent yet specific treatment that is able to facilitate tumor eradication whilst sparing normal cells is considered the “holy grail” in cellular therapy. The majority of CAR T cell target antigens to date are those overexpressed on tumor cells but also expressed at lower levels on normal tissues. While such antigens, for example GD2, Lewis Y and CEA may serve as relatively safe targets for CAR T cells (provided that their expression levels on healthy vs. malignant cells are sufficiently distinguishable) (41), this kind of differential expression unfortunately does not exist for most myeloid antigens, hence prompting the search for truly tumor-specific antigens. A newly formed antigen that results specifically from a disease-causing or disease-associated mutation would be the preferred target for CAR T cells as it should be expressed by malignant cells but not healthy cells (42). The Cancer Genome Atlas Research Network has conducted a comprehensive study to examine the mutational composition of *de novo* AML, and a number of recurrent mutations contributing to leukemogenesis have been identified (43, 44). AML genomes are amongst those with the lowest mutational burden, and presumably present very few neoantigens (45). Nonetheless, there are some neoantigens that have been described in AML, including mutations in the metabolic enzymes IDH1 and IDH2, which are present in ~20% of *de novo* AML cases (46, 47). Immune recognition of these IDH1/2 mutants have been demonstrated, suggesting endogenous processing and presentation of the mutant epitopes (48). In addition, mutation in NPM1 gene is also one of the most frequent genetic alterations in patients with AML, with up to 60% patients with normal cytogenetics reportedly having NPM1 mutation (49). Similar to IDH1/2 mutants, the NPM1 mutant epitopes have also been shown to be immunogenic, inducing both CD8^+^ and CD4^+^ T cell responses (49). The existence of such neoantigens in AML, albeit at relatively low frequency, makes them an attractive target for T cell therapy. However, the proteins encoded by these disease-associated mutations are expressed intracellularly, and therefore are not accessible to the CAR.

Dysregulated splicing can also be a source of neoantigens, if it results in alternative isoforms that are distinguishable from their wildtype counterpart. A recent study reported that about a third of expressed genes in AML undergo differential RNA splicing. This may result in splice variants and therefore potential neoantigens (50). In a subsequent study from the same group, expression of two novel splice variants, one for Flt3 and another for NOTCH2, was reported to be found in 50 and 73% of AML cases, respectively, but was absent in healthy donors (51). Additionally, another AML-specific isoform was described to arise from CD44, called the CD44v6 variant. In one study, more than 60% of the AML cases showed expression of CD44v6, which was not present in normal HSPC. Unlike other disease-associated mutations that are expressed intracellularly, CD44v6 is expressed on the cell surface, making it accessible to the CAR. Indeed, CAR T cells targeting the CD44v6 variant were generated and reported to exert robust anti-tumor responses against primary AML cells, apparently whilst preserving the HSPC compartment (52).

Although CARs can only recognize antigens expressed on the cell surface, notably a recent innovative study by Rafiq et al. has demonstrated that generating a CAR that recognizes an intracellular antigen is in fact possible. In this study, a T cell receptor (TCR)-mimic CAR, whose specificity was directed toward the intracellular onco-protein WT1 that was presented on the cell surface in the context of MHC, therefore targeting a peptide-MHC complex (53). This study provided a proof-of-concept for the possibility of expanding the range of CAR recognition beyond extracellular antigens, and that intracellular tumor-specific mutant epitopes could be harnessed for CAR T cell therapy. Although not yet tested clinically in the context of CAR T cells, the clinical use of WT-1-directed T cells was recently reported in a study by Chapuis et al. in a TCR-based adoptive therapy setting (54). This study sought to investigate whether AML relapse risk following HSCT could be decreased by prophylactically giving the patients WT-1-directed T cells. Remarkably, all twelve patients who received WT-1-directed T cells post HSCT had 100% relapse-free survival rate at a median of 44 months post transfer, which was significantly higher compared to the 54% relapse-free rate observed in the accompanying comparative group of 88 patients who had similar risk AML (54).

### Challenges in Manufacturing CAR T Cells

The manufacture of CAR T cells in patients with active AML may prove to be problematic. It has been previously reported that AML cells secrete soluble factors that inhibit T cell proliferation (55). A study from our group has demonstrated that the presence of AML blasts could indeed be detrimental to the ability of T cells to expand and thus negatively affect CAR T cell production (56). In our recent clinical trial experience for the treatment of R/R AML, we encountered some CD123-CAR T cell manufacturing problems, resulting in ~40% of the planned doses failing to be administered (17). It is important to note however, that another group at the City of Hope did not encounter similar difficulties (22), the difference of which may be attributable to the different CAR T cell production methods used—our group used mRNA electroporation to deliver the CAR, whereas the other group used lentiviral transduction. Another potential issue that may give rise to challenges in the generation of CAR T cells is the fact that patients with AML that are candidates for CAR T cell therapy will have most likely undergone heavy and intense treatments that may make it harder to obtain good quality T cell product for CAR T cell manufacture. It is possible that careful selection of patients may potentially address this issue, perhaps by selecting patients relatively early in their treatment course but who are likely to be chemo-refractory based on accepted prognostic markers (57). Alternatively, many groups are now evaluating the use of allogeneic product from healthy donors as the source for CAR T cell manufacture, though it comes with its own risks of graft vs. host disease (GvHD) and/or rejection of the transferred CAR T cells (58).

A number of solutions to circumvent the risks associated with allo-CAR T cell therapy have been examined. For instance, intensifying the pre-conditioning regimen may be sufficient to prevent CAR T cell rejection. In an attempt to alleviate GvHD, data from various pre-clinical studies point to a new approach involving genetic disruption of TCR from CAR T cells (59, 60). As a result, this strategy has entered the clinic, and in 2017 it was reported that two children with R/R B-ALL were successfully treated with allogeneic TCR-deficient CD19-CAR T cells (61). Additional clinical trials using these so-called universal CAR T cells are ongoing (*clinicaltrials.gov identifier: 03166878, 03229876*). While this approach is promising, it is important to note that there still exists a risk of GvHD caused by even very small percentage of TCR^+^ cells that may remain in the T cell population (62). Optimization of TCR deletion during CAR T cell manufacture would therefore be a crucial step for the clinical application of universal CAR T cells.

Numerous studies have also assessed potential alternatives to CAR T cells that may help reduce the risk of GvHD. To date, most of the CAR T cells used in the clinic are made from unselected T cells, consisting primarily of αβ T cells. An attractive option is to select a T cell subset that is less likely to induce GvHD for the manufacture of CAR T cells. As such, studies have explored the possibility of utilizing γδ T cells, which unlike αβ T cells, are not alloreactive and do not induce GvHD (63). The incorporation of CAR into γδ cells was first reported in 2004 using a first-generation CAR specific for GD2. The study reported that elevated antigen-specific tumor reactivity was observed (64). Although γδ cells represent only a small percentage of up to 5% circulating T cells, in another study using GD2-directed CAR T cells, it was demonstrated that γδ cells (both Vδ1 and Vδ2 subsets) could be successfully expanded and transduced to sufficient numbers for use in clinical studies. CAR expression by these γδ cells was shown to augment their innate cytotoxicity in a CAR-antigen specific manner (65). The clinical use of CAR γδ cells has not been reported to date, however there have been numerous clinical trials evaluating the safety and activity of adoptively transferred Vδ2 T cells (with no CAR manipulation) for multiple cancer types (66). Accruing data suggest that γδ cells may offer a safer alternative to the conventional αβ T cells for allogeneic use of CAR T cells. However, there may be differential anti-tumor and pro-tumor functions in different γδ cell subtypes (67), suggesting that careful selection of γδ T cell subsets to use for CAR manipulation will be of high importance.

Alternatively, other effector cells such as NKT and NK cells may also be used for adoptive therapy to mitigate GvHD risks. CAR incorporation into NKT cells has been done in some pre-clinical studies against several tumor antigens (68, 69). Similarly, NK cells engineered to express CAR (CAR-NK) have also been tested in various *in vivo* models, using both primary human NK cells and the NK-92 cell line, with the goal of augmenting their cytotoxic function while exploiting their innate mechanisms for detecting and killing cancerous cells (70, 71). A number of CAR-NK cells are currently being tested in phase I/II clinical trials, mostly targeting CD19 but also other antigens including CD7, CD33, MUC-I, and Her2 (72). Of note, insufficient numbers of NK cells that can be used for therapy, as well as their lack of *in vivo* persistence, have long posed a significant limitation on NK cell-based therapeutic approaches. A phase I/II dose escalation study of CAR-NK cells has been initiated to address this issue in patients with B-cell malignancies (*clinicaltrials.gov identifier: 03056339*). At present, increasing NK cell expansion and persistence remains an active area of study. Encouragingly, a recent publication reported that inclusion of IL-15 transgene can lead to successful expansion of cord blood-derived CAR123-NK cells (71).

### Immunosuppression Induced by AML

**Figure 1:**
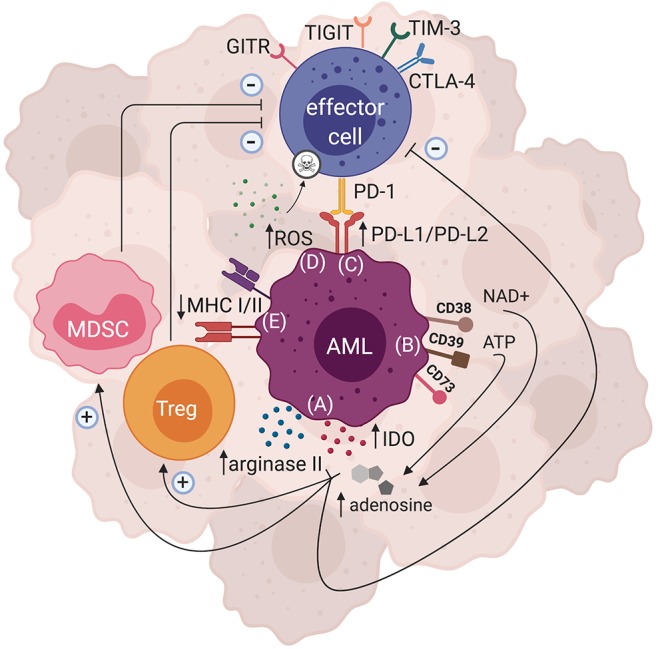
Mechanisms of AML-induced immunosuppression. There are several immunosuppressive pathways that have been described for AML. Both direct and indirect mechanisms can ultimately lead to AML immune escape. **(A)** Enzymes such as arginase II and IDO can be expressed by AML blasts, leading to production of metabolites that hinder effector cell function and proliferation, while polarizing the tumor microenvironment to become more immunosuppressive by favoring Treg and MDSC expansion. **(B)** AML blasts can express ectonucleotideases such as CD38, CD39, and CD73 that are involved in the breakdown of ATP and NAD+ to adenosine, which subsequently dampens effector cell function and enhances the activity of immunosuppressive cells. **(C)** Inhibitory ligands such as PD-L1 and PD-L2 can be expressed by AML blasts, and upon binding with their cognate PD-1 receptor may lead to effector cell suppression. Expression of other inhibitory receptors such as GITR, TIGIT, TIM-3, and CTLA-4 have also been shown in AML. **(D)** AML blasts are able to produce large amounts of ROS that subsequently trigger apoptosis of effector cells. **(E)** AML blasts can also downregulate their MHCI and/or MHCII expression, thus impairing their antigen presentation resulting in immune evasion.

Though AML has long been known to be immune-responsive, as highlighted by the graft vs. leukemia effects following allo-HSCT (2), accumulating evidence has suggested AML to also be an immunosuppressive and/or immune-evasive disease (73). A wide range of mechanisms by which AML causes immune-evasion have been reported, the most compelling of which is acquired partial uniparental disomy (UPD) of chromosome 6p, which has been reported in AML patients relapsing after haploidentical HSCT. UPD of chromosome 6p, where the human leukocyte antigen (HLA) is encoded, leads to the loss of the mismatched HLA haplotype, rendering the host's leukemia unrecognizable by donor T cells (74, 75). Notably, downregulation of MHC class I and/or MHC class II has also been observed in AML patients (76, 77), and a recent study by Christopher *et al*. reported MHC class II downregulation to be associated with AML relapse after transplantation (77). While these specific mechanisms are not likely to be relevant for post-CART cell relapse, they are reminiscent of recent observations regarding loss of the target epitope after anti-CD19 CART cell therapy (78). Various other immunosuppressive mechanisms have also been described for AML, most of which are based on *in vitro* and pre-clinical observation. It is important to identify the immunosuppressive axes responsible for AML immune evasion in order to uncover potential therapeutic targets and thus develop appropriate therapies. Discussed below are some of the known pathways through which AML can induce immunosuppression (Figure 1).

#### Secretion of Immunosuppressive Soluble Factors

T cell dysfunction observed in the presence of AML blasts may be attributable, in part, to the immunosuppressive soluble factors secreted by AML blasts (55, 79, 80). For example, the activity of arginase II enzyme has been shown to be significantly elevated in the plasma of patients with AML in comparison to normal donors. When T cells are cultured with the plasma from these patients, a decrease in T cell proliferation was observed, and this could be rescued by replacement of arginine (80). In this study, the production of arginase II by AML blasts was reported to not only directly dampen T cell activity, but also polarize the monocyte population toward an immunosuppressive phenotype (80). Arginase inhibitors are in clinical development for advanced solid malignancies (*clinicaltrials.gov identifier:* 02903914) (81).

Another enzyme that has attracted interest in AML and numerous other malignancies is indoleamine 2,3 dioxygenase (IDO). This enzyme is involved in the oxidation of tryptophan to *N*-formylkynurenine, and is expressed at high levels in activated dendritic cells (DCs) and macrophages. The breakdown of tryptophan is known to hinder T cell proliferation and differentiation (82). In addition, the activity of IDO enzyme has also been reported to promote Treg conversion and enhance their immunosuppressive function (83–85). Increased levels of kynurenine are detected in patients with AML and correlated with diminished overall survival (86). AML blasts may be the source of the elevated levels of kynurenine seen in patients, given that they express IDO, both constitutively or after IFNγ exposure. This is further supported by a finding that shows direct correlation between higher Treg population in patients with AML and the blast IDO expression (87). Overall, the immunosuppressive effects of IDO make it an appealing therapeutic target, and efforts to block this pathway have led to the development of IDO inhibitors that are currently undergoing clinical testing in various types of malignancy including AML (*clinicaltrials.gov identifier:* 02835729) (88). In the context of CAR T cell therapy, several approaches that limit IDO expression and activity have been shown to improve CAR T cell efficacy in pre-clinical models. IDO1 gene is a putative target of miR-153, and overexpression of miR-153 was reported to suppress IDO expression and ultimately led to better CAR T cell efficacy in an *in vivo* model (89). Another study showed that the use of lymphodepleting drugs frequently used for pre-conditioning regimen, cyclophosphamide and fludarabine, downregulated IDO expression and augmented CD19-CAR T cell activity *in vivo* (90). Together, these observations support the combinatorial potential of IDO inhibition and CAR T cell therapy, and warrant further investigations including in myeloid malignancies.

#### Immunosuppressive Cellular Compartment

Cancer cells are able to recruit and/or promote the expansion of immunosuppressive cells including Tregs and myeloid-derived suppressor cells (MDSCs) (91). Increased Treg frequency has been reported in peripheral blood and bone marrow of patients with AML, and seems to correlate with poor prognosis (92, 93). AML cells can also produce large amounts of adenosine, as a result of adenosine triphosphate (ATP) or nicotinamide adenine dinucleotide (NAD+) breakdown mediated by ectonucleotideases that are expressed not only by the AML blasts, but also Tregs. The resulting adenosine accumulation leads to suppression of effector T cell function as well as augmentation of Treg activity (94). MDSCs are another immunosuppressive immune cell subset that are increased in patients with AML. In an *in vitro* co-culture assay, expansion of MDSCs was shown to be induced by both primary AML cells and AML cell lines in a contact-dependent manner. It was further identified that MUC-1 oncoprotein was responsible for the observed MDSC expansion (95). AML blasts themselves may possess many features of MDSCs, as discussed in a recent review (96). Notably, AML blasts, specifically the monocytic subtype, have been reported to directly suppress T cell anti-tumor responses by inducing large amounts of reactive oxygen species (ROS), subsequently triggering T cell apoptosis (97).

Several potential solutions could address the issue of abundant immunosuppressive cells in the context of CAR T cell therapy. Although not yet tested in AML, adenosine-mediated immunosuppression could be alleviated by combining CAR T cell therapy with antagonists targeting the adenosine receptor A_2A_R, as shown in a pre-clinical model for solid tumors (98). In addition, a number of clinical trials have been initiated in an attempt to reduce the abundance and recruitment of MDSCs in multiple malignancies by using small molecules such as liver-X nuclear hormone receptor (LXR) agonist and chemokine inhibitors (99). Given the T cell-inhibiting role of MDSCs, combining CAR T cells with small molecules that can deplete the MDSC population may result in synergistic effects. Indeed, at least in an *in vivo* model, MDSC depletion was shown to increase CAR T cell anti-tumor effects (100). In addition to impairing CAR T cell function, AML-induced immunosuppression is likely to also present a challenge for CAR T cells to persist *in vivo*. Though not yet tested specifically for AML, several strategies that have been shown to improve CAR T cell expansion and persistence include expression of IL-15 (101) or IL-18 transgene (102), addition of exogenous IL-7, IL-15, and/or IL-21 cytokines during CAR T cell *ex vivo* expansion (103, 104), and many others that are currently being developed to skew CAR T cell phenotype toward a stem cell/ central memory phenotype. Additionally, in an attempt to further improve CAR T cell efficacy, approaches that are able to boost CAR T cell activation, such as the use of immune agonists (100, 105) or co-stimulatory cytokines (106, 107) may lead to improved overall efficacy.

#### Upregulation of Inhibitory Ligands and Receptors

The roles of immune inhibitory ligands and their receptors have been extensively studied in a wide range of cancers. Decades of work invested in studying these immune checkpoints led to widespread clinical use of several monoclonal antibodies (mAbs) that block specific signaling pathways such as CTLA-4 and PD-1 (108, 109). The remarkable success of these mAbs have been highlighted by the FDA approval of α-PD-1, α-PD-L1, and α-CTLA-4 mAbs for a number of cancers, and more recently by the Nobel Prize in physiology and medicine to Tasuku Honjo and James Allison (110). Despite the overwhelming enthusiasm with which checkpoint inhibitors are being trialed in solid cancers, there are only limited data to date that support the role of a single inhibitory pathway in mediating AML immune-evasion. This discrepancy is thought to be due to, in part, the fact that AML has among the lowest mutational burdens in human cancer (111), thus presumably fewer neoantigens recognized by T cells.

In an autologous culture system, blockade of CTLA-4 was shown to augment the activity and expansion of AML-reactive T cells (112). To date, the most encouraging clinical data that supports the importance of CTLA-4 pathway in AML comes from a phase I trial exploring the use of ipilimumab in patients with hematological malignancies relapsing post allo-HSCT. In this study, the five patients achieving complete remission had AML. An interesting observation was that four of these patients had extramedullary disease (113).

The PD-1:PD-L1/PD-L2 axis has been investigated in AML, although it is unclear whether PD-L1 is consistently overexpressed on primary AML blasts (114, 115). PD-L1 may be upregulated upon exposure to inflammatory stimuli, such as toll-like receptor (TLR) ligands and IFNγ (116), and expression of PD-L1 and/or PD-L2 by AML blasts may be associated with poor prognosis (117). Expression of the PD-1 receptor on T cells has been described in patients with AML at relapse (117), and in an *in vivo* model, PD1^−/−^ mice inoculated with AML had slower disease progression compared to PD-1-sufficient mice (118). As noted earlier, given the limited clinical efficacy of checkpoint inhibitors in AML as a single agent, researchers are now looking into combinatorial approaches with other anti-cancer modalities such as the hypomethylating agent, azacitidine, which is known to upregulate PD-1 and PD-L1 expression (*clinicaltrials.gov identifier: 02775903, 03092674*) (119). Other checkpoint pathways of interest in AML include T cell immunoglobulin and immunoreceptor tyrosine-based inhibitory motif domain (TIGIT), T cell immunoglobulin domain and mucin domain 3 (TIM-3), and glucocorticoid induced tumor necrosis factor receptor related protein (GITR) (120–122).

CAR T cell combination with checkpoint inhibitors, either exogenously administered (123), or via genetic engineering of the CAR T cells themselves to synthesize the checkpoint blocking antibodies (124, 125), has been tested in pre-clinical models against several cancer types. These combination strategies have resulted in promising pre-clinical data, and more recently encouraging preliminary clinical data (126–128), which ultimately paved the way for their clinical translation. This has been highlighted by the numerous clinical trials currently ongoing to evaluate the safety and efficacy of the combined CAR T cell and ICB therapy, particularly for lymphomas and various solid tumors (129). As previously noted, the underwhelming effects of checkpoint inhibition in AML may be due to the low mutational burden nature of AML and thus the low frequency of AML-reactive T cells (111). We postulate that this therefore makes tumor-targeted CAR T cells an ideal partner for combination with checkpoint inhibitors in AML.

## Conclusion and Future Directions

AML is an aggressive disease that, if not completely eliminated at first attempt, becomes resistant to further treatments. Robust therapies that eliminate all leukemia cells including putative leukemic stem cells are therefore needed to achieve deep and durable remissions. CAR T cell therapy has proven to be a potent immunotherapy weapon and is already being tested in the clinic for AML (and potentially other myeloid malignancies including MDS, MPN, and CML) (Table 1). However, there are a number of barriers that limit the full therapeutic potential of CAR T cells. First, there are no truly leukemia-specific cell-surface antigens that could be used as targets for CAR T cells. AML antigens are frequently shared by normal HSPCs or their progeny. Additionally, the manufacture of CAR T cells itself may also be a challenge in patients with active AML, potentially due to inhibition of T cell expansion by AML blasts, or prior exposure to T cell-damaging chemotherapy. Further, it is increasingly apparent that AML is a heterogeneous and complex disease capable of evading the immune system by means of various immunosuppressive mechanisms. Therefore, although CAR T cell therapy for AML is already in clinical trials, we are facing challenging yet surmountable obstacles to ensure the safe and effective use of CAR T cells for AML. As discussed in this review, there are many potential avenues to reduce toxicity to healthy tissues while maintaining, and even augmenting, the full therapeutic potential of CAR T cells. We strongly advocate for careful, rationally-designed clinical trials that are informed by a comprehensive and clear-eyed understanding of the AML—effector cell—microenvironment axis in order to advance toward a shared goal of successful AML immunotherapy.

**Table 1:** CAR T cell trials in myeloid malignancies currently recruiting.

**Disease**	**Interventions**	**Identifier ID**	**Phase**	**Location**
AML	CD123/CLL1 CAR T cells	NCT03631576	II/III	Fujian Medical University Union Hospital, China
	CLL-1, CD33 and/or CD123 CAR T cells	NCT04010877	I/II	Shenzhen Geno-Immune Medical Institute, China
	CD123 CAR T cells	NCT03796390	I	Hebei Yanda Ludaopei Hospital, China
	CD123 CAR T cells	NCT03585517	I	Xian Lu, China
	Muc1/CLL1/CD33/CD38/CD56/CD123 CAR T cells	NCT03222674	I/II	Zhujiang Hospital of Southern Medical University, Yunnan Cancer Hospital, Shenzhen Geno-immune Medical Institute, China
	CD38/CD33/CD56/CD123/CD117 /CD133/CD34/Mucl CAR T cells	NCT03473457	N/A	Southern Medical University Zhujiang Hospital, China
	CD123 CAR T cells expressing EGFRt		I	Fengtai District, China
	CD44v6 CAR T cells	NCT04097301	I/II	IRCCS San Raffaele, IRCCS Ospedale Pediatrico Bambino Gesù, Italy
	CD33 CAR T cells	NCT03971799	I/II	The Children's Hospital of Philadelphia, USA
	Universal CD123 CAR T cells	NCT03190278	I	H. Lee Moffitt Cancer Center, Dana-Farber Cancer Institute, Weill Medical College of Cornell University, MD Anderson Cancer Center, USA
	CD123 CAR T cells	NCT04014881	I	Union Hospital, Tongji Medical College, Huazhong University of Science and Technology, China
	CD123 CAR T cells	NCT03556982	I/II	307 Hospital of PLA, China
	CD123 CAR T cells expressing EGFRt	NCT02159495	I	City of Hope Medical Center, USA
	CD123 CAR T cells	NCT03766126	I	University of Pennsylvania, USA
AML/MDS	CD33/CD38/CD56/CD117/CD123/CD34 /Muc1 CAR T cells + Eps8 or WT1 peptide specific dendritic cell	NCT03291444	I	Zhujiang Hospital, Southern Medical University, China
	NKG2D CAR T cells	NCT03018405	I/II	USA and Belgium
AML/MDS/MPN	CD123-CD33 cCAR T cells	NCT04156256	I	Chengdu Military General Hospital, Peking University Shenzhen Hospital, China
	CLL1-CD33 cCAR T cells	NCT03795779	I	The General Hospital of Western Theater Command, Peking University Shenzhen Hospital, China
CML	IL-1RAP CAR T cells	NCT02842320	N/A	Hôpital Nord Franche-Comté, Centre Hospitalier Régional Universitaire de Besançon, CHU de Dijon, CHI de Haute-Saône, France

*Information available from www.clinicaltrials.gov using search term “CAR” and “AML” or “MDS” or “MPN” or “CML” in January 2020. AML: acute myeloid leukemia; CAR, chimeric antigen receptor; CML, chronic myeloid leukemia; MDS, myelodysplastic syndrome; MPN, myeloproliferative neoplasms; EGFRt, epidermal growth factor receptor truncated*.

## Author Contributions

SM and SG wrote the manuscript.

## Conflict of Interest

Research Funding: Novartis, Tmunity Therapeutics, Carisma Therapeutics. Stock and Ownership: Carisma Therapeutics. Consulting: Aileron, Fate, Sensei. The remaining author declares that the research was conducted in the absence of any commercial or financial relationships that could be construed as a potential conflict of interest.
